# Bioconversion of Digestate, Pig Manure and Vegetal Residue-Based Waste Operated by Black Soldier Fly Larvae, *Hermetia illucens* L. (Diptera: Stratiomyidae)

**DOI:** 10.3390/ani11113082

**Published:** 2021-10-28

**Authors:** Teun Veldkamp, Klaas van Rozen, Hellen Elissen, Piet van Wikselaar, Rommie van der Weide

**Affiliations:** 1 Wageningen Livestock Research, De Elst 1, 6700 AH Wageningen, The Netherlands; piet.vanwikselaar@wur.nl; 2 Wageningen Plant Research, Edelhertweg 1, 8219 PH Lelystad, The Netherlands; klaas.vanrozen@wur.nl (K.v.R.); hellen.elissen@wur.nl (H.E.); rommie.vanderweide@wur.nl (R.v.d.W.)

**Keywords:** bioconversion, biowaste, black soldier fly larvae, digestate, manure

## Abstract

**Simple Summary:**

Waste management strategies which favour the value of the organic waste instead of its disposal should be developed. One of the insects able to convert biowaste into high valuable protein and fat is the black soldier fly (*Hermetia illucens*). This article describes a preliminary assessment of the larval growth and biowaste conversion of *Hermetia illucens* larvae reared on digestate (biogas slurry), pig manure and vegetal residue-based waste in order to select most promising organic waste for further research. Larval growth was highest when reared on “Swill” (catering waste) and was almost twice as high as on the reference substrate “Chicken feed”. Other biowaste sources tested in this experiment with potential for further research were “Pig manure liquid mixed with chicken feed” and “Pig manure solid”. Most promising organic waste sources from this experiment were selected for a follow-up experiment to study the effect of different combinations of organic waste on biowaste conversion by *Hermetia illucens* larvae.

**Abstract:**

Insects can play an important role to upgrade waste streams into high-grade proteins and fats as food and feed ingredients or non-food products. The aim of this research was to assess the feasibility to use waste streams with a low value for direct application as animal feed as substrates to grow BSF larvae in terms of larval growth rate, waste reduction index, and efficiency of conversion of ingested feed. The growth of black soldier fly (BSF), *Hermetia illucens* larvae and conversion of biowaste was assessed in triplicate in biowaste substrates: chicken feed (CF; reference diet), pig manure solid (PMS), Betafert^®^ solid (BTFS), swill (SW), olive pulp (OP), pig manure liquid mixed with chicken feed (PMLCF), and silage grass (SG). Per kilogram fresh substrate 2500 starter (8-days-old, second instar) larvae were incubated in 21 plastic containers (75 × 47 × 15 cm). The BSF larvae were fed according to a batch feeding system. Highest growth rate was found in larvae reared on SW (13.4 mg/d). Larval growth rate was even higher than in larvae reared on the reference substrate CF (7.2 mg/d). Growth rate in larvae reared on PMLCF (7.3 mg/d) did not differ from CF, whereas growth rate of larvae reared on PMS (3.2 mg/d) was lower than on CF. Growth rate of larvae reared on BTFS, OP and SG was very low (0.6, 0.2 and 0.7 mg/d, respectively). Waste Reduction Index (WRI) was highest on SW (11.3), followed by PMLCF (9.3), and both were higher than WRI on CF (8.5). Waste Reduction Index further decreased in descending order from PMS, SG, BTFS to OP (7.6, 4.0, 2.9 and 1.7, respectively). The Efficiency of Conversion of Ingested substrate (ECI) was highest on SW (0.31), followed in descending order by PMLCF, CF and PMS (0.25, 0.21 and 0.18, respectively). The substrates OP, BTFS and SG (0.16, 0.15 and 0.14, respectively) resulted in a lower ECI than other substrates. Highest CO_2_ and lowest NH_3_ concentrations were found above substrates with the highest larval growth performances. This study showed that BSF larvae can be reared on different biowaste substrates; the growth rate of the larvae was extremely high on SW. The effects of chemical composition and physical properties of the substrates on larval growth and gas emissions should be further considered.

## 1. Introduction

Roughly one-third of food produced for human consumption is lost or wasted globally, which amounts to about 1.3 billion tons per year [1]. In the European Union, despite the production of 89–100 million tons of biowaste every year, only around 3 million tons are recycled as livestock feed due to consumer and farmer concerns related to food safety and disease control [2]. Waste management strategies, which favour the value of the organic waste instead of its disposal, should be developed. Animals may be used to provide a significant contribution to waste treatment, in addition to the well-known aerobic bacterial and anaerobic degradation of biowaste during composting and biogas production. Biowaste that cannot be used directly as feed ingredient may be fed as a first step to insects to upgrade this waste into high-grade proteins and fats as food and feed ingredients or non-food products. Biowaste that is suitable and allowed to feed directly to livestock animals should be fed directly, otherwise sustainability of the production chain is not improved. Insects associated with manure and biowaste can play a key role for the sustainable valorisation of biowaste streams [3]. One of the insects able to convert biowaste into high valuable protein and fat is the black soldier fly (BSF; *Hermetia illucens* L.) [4,5].

The conversion potential of biowaste by BSF larvae is heavily dependent on the nature of substrates provided to these larvae [6,7,8,9]. These authors implicate that the concentration and ratio of protein and carbohydrates affect development and nutrient composition of the larvae. Moisture content in the substrates also affects development and harvesting of the larvae. Residue from food waste containing 70% or 75% moisture could be effectively separated from insect biomass by using a 2.36 mm sieve but this was not feasible for residue from food waste with 80% moisture [10]. The presence of excessive moisture content in the waste can hinder its decomposition rate and the resulting residue with a sticky consistency is known to result in a clumpy material difficult for further processing [11]. Texture of the substrate is also important for mobility of the larvae and accessibility to nutrients, but so far literature is scarce on this issue. This study offers a preliminary assessment of the development and growth of *Hermetia illucens* larvae reared on digestate (biogas slurry), pig manure and vegetal residue-based waste varying in dry matter content and texture. The waste streams were selected, based on the current economic value, as those are currently used as a fermentation source or offered for incineration, with the aim to assess the feasibility to use these waste streams as substrates to grow BSF larvae in terms of larval growth rate, waste reduction index, and efficiency of conversion of ingested feed. Converting these substrates into high-grade insect products will highly contribute to a circular and climate-neutral society, and after this study on development and growth of *Hermetia illucens* larvae, the most promising waste streams will be selected for further optimizing by mixing in follow-up experiments.

## 2. Materials and Methods

Black soldier fly (BSF; *Hermetia illucens*) larvae were obtained from the commercial insect rearing company Bestico B.V. (Berkel en Rodenrijs, The Netherlands). The larvae were 8 days of age at arrival. In total, seven rearing substrates were tested: Chicken feed (CF; reference diet, broiler grower diet, Agri Retail B.V.), pig manure solid (PMS), Betafert^®^ solid (BTFS), swill (SW), olive pulp (OP), pig manure liquid mixed with CF (PMLCF), and silage grass (SG). Chicken feed (Agruniek Rijnvallei Voer BV, Wageningen, The Netherlands) was used as a reference diet (19.8% crude protein, 5.3% fat, 6.2% crude fiber, and 5.2% crude ash). Chicken feed was also used as a reference diet in other experiments [12,13]. Pig manure solid were sow faeces provided by Van Beek SPF Varkens B.V. (Lelystad, The Netherlands). Betafert^®^ is digestate (biogas slurry) from the SuikerUnie biomass digester in Dinteloord (The Netherlands) and was delivered by AgriBioSource Europe B.V. (Nijmegen, The Netherlands). The digestate is produced from sugar beet residues (beet pulp, leaves and root tips) after solid-liquid separation. Catering swill was provided by Tekagroup BV (Barneveld, The Netherlands) and mainly consisted of pasta, potatoes and vegetables (no animal products). Olive pulp (also called olive pomace, olive mill waste or olive cake) is what remains after olive oil extraction (pulp, skins, kernels and water) and was obtained from ACRRES (Lelystad, The Netherlands). Pig manure liquid was pig slurry (mixed faeces and urine) (same source as the solid pig manure) and was mixed with CF. Grass silage was nature grass obtained from Staatsbosbeheer (The Netherlands) fermented by ensiling at ACRRES (Lelystad, The Netherlands).

Substrates were obtained one week prior to the start of the rearing cycle and kept at 4 °C until further use. Different quantities of substrates were provided in the containers to realise a substrate layer of approximately 5 cm (Table 1). A batch feeding strategy was applied, which means that the substrates were placed in the plastic containers one day prior to placing the larvae at the start of the experiment. This allows the substrates to heat up until the start of the experiment. The substrates were not pre-treated other than chicken feed (CF) and olive pulp (OP) substrates were mixed with water (1:1 *w*/*w*). Pig manure liquid was mixed with CF (1:1 *w*/*w*). Particle size of the different substrates was not recorded. On top of each substrate, 2500 starter (8 days-old) larvae per kilogram (wet) substrate were incubated in 21 plastic containers (75 × 47 × 15 cm) (Table 1). Each substrate was tested in triplicate in a climate chamber. The temperature was 30.1 ± 0.5 °C and relative humidity (RH) was 46.4 ± 3.7% at day 1 and 60.6 ± 1.6% from day 2 until the end of the experiment. The photoperiod was 0:24 h light:dark. The plastic containers were randomly arranged in three piles with vents between the containers. On day 3, water (450 mL/container) was added to the containers with PMS because from visual observation it was concluded that this substrate was too dry for consumption by larvae. The duration of the experimental period for CF, PMS, SW and PMLCF was 8 days and the duration for BTFS, OP and SG was 11 days. Experimental durations varied according to the substrate consumption estimated from the 21 containers and/or with the appearance of 10% prepupae. 

Dry matter content of the substrates at the start of the experimental period and of the substrate/frass and BSF larvae at the end of the experiment were determined by oven-drying for 48 h at 105 °C. Mean individual start wet weight of the larvae (11.43 mg) was determined from three samples of ca. 400 larvae. The containers were placed in three boxes (120 × 100 × 60 cm) mostly at random but not completely as the containers with highest moisture content were placed at the bottom to avoid escaping larvae falling down in containers below them. Duplicate samples of substrate, frass and larvae were collected from the remaining substrate in each container and larvae were separated from substrate and frass, counted and weighed. The number of larvae collected in these samples varied between 45 and 756 and mean larval wet weight was calculated from these samples. Survival of the larvae could not be determined because removing the top-layer of the substrate/frass included an unknown number of larvae. The method used to calculate the larval growth rate (g/d) was according to [14]:

*Larval growth rate* = (*final larval average weight* − *initial larval average weight*)/*number of days of the trial*. (1)

To evaluate larval efficiency in consuming and metabolizing the growing substrates, the total final biomass (larvae + prepupae) and the residual substrates were weighted. Waste reduction index describes the larval ability to reduce feeding substrates, taking into account the number of days the larvae were fed on the substrates; therefore, higher values show a greater ability to reduce the organic matter. The conversion efficiencies are based on dry matter because efficiencies on a fresh matter basis can be obscure, as considerable variation is present in the dry matter contents of the substrates. Waste reduction index (WRI) and the efficiency of conversion of the ingested feed (ECI) were calculated for the determination of the waste consumed by the larvae and the conversion efficiency of the substrates into insect biomass. The following indexes were calculated:

*Waste reduction index* (*WRI*) = ((*W* − *R*)/*W*)/*days of trial* (*d*) × 100 (2)

where *W*= total amount of substrate provided; *R* = residual of the substrate, and

*Efficiency of conversion of the ingested food* (*ECI*) = *B*/(*W* − *R*) (3)

where *B* = total larval + pupal biomass at the end (g); *W* = total amount of substrate provided; *R* = residual of the substrate.

CO_2_ and NH_3_ gas concentrations in the climate chamber were measured manually at different times per day. CO_2_ and NH_3_ concentrations were also measured at substrate level by use of plastic buckets and a calibrated Innova 1412A-5, a photo-acoustic infrared detection method from LumaSense Technologies. The air quality sensor detects gases through chemical reactions, producing a current that is directly proportional to the concentration of target gas present and shows good responses to various gas concentrations over a wide range of ambient conditions. The buckets were placed on the substrates in the containers and gases were measured three times during a period of 30 min at day 8. 

Dry matter content, larval growth and bioconversion data were analysed by analyses of variance using the statistical program Genstat, 19th Edition (https://www.vsni.co.uk/software/genstat/, accessed on 17 November 2020) [15] in a randomized block design. Each rearing box served as a statistical unit and a pile of seven plastic containers was considered as block. Data are presented as the mean and considered significant if *p* ≤ 0.05 and a tendency if 0.05 < *p* ≤ 0.10.

## 3. Results

At the end of the experimental period containers with CF still contained residual substrate in addition to the frass. Rearing on PMS resulted in a lot of dry granular frass with dry non-converted crusty substrate material on top. Rearing on BTFS resulted in a dry top-layer, while frass was found at the bottom of the containers with BTFS. SW was completely converted into larval biomass and frass and no substrate was left. The larvae were sticky, i.e., the larvae were covered with an oily substance. Rearing on OP resulted in a solid granular top-layer while some frass was found at the bottom of the containers with OP. Rearing on PMLCF resulted in an almost similar remaining substrate as CF, but with a solid top-layer. Rearing on SG resulted in a dry top-layer with a moist bottom-layer. Dry matter concentrations of the substrates at the start and end of the experiment are presented in Table 2.

Initial dry matter content of OP was highest, subsequently followed by CF, PMLCF, PMS, SG, BTFS and SW. During the experiment, dry matter content of the substrates increased. Highest final dry matter content was determined in CF and PMLCF, subsequently followed by OP and PMS, SW, SG and BTFS. Dry matter content of larvae increased along with higher larval growth rate during the trial. The highest dry matter content was found for larvae on SW, subsequently followed by CF and PMLCF, OP, PMS, BTFS and SG. Dry matter content of larvae on BTFS, PMS and SG was very low.

Highest growth rate was found in larvae reared on SW (Table 3). Larval growth rate was significantly higher than in larvae reared on the reference substrate CF and PMLCF. Growth rate of larvae reared on PMLCF did not significantly differ from CF, whereas growth rate of larvae reared on PMS was significantly lower than on CF. Growth rates of larvae reared on BTFS, OP and SG were very low and significantly lower than other substrates. Waste Reduction Index (WRI) was highest on SW followed by PMLCF, and both were higher than WRI on CF. Waste Reduction Index decreased further in descending order from PMS, SG, BTFS to OP. The Efficiency of Conversion of Ingested substrate (ECI) was highest on SW followed by PMLCF, CF and PMS. The substrates OP, BTFS and SG resulted in a lower ECI than other substrates. 

Gas concentrations were measured in the entire climate chamber, which means that gas concentrations cannot be related to one of the substrates separately. 

**Figure 1:**
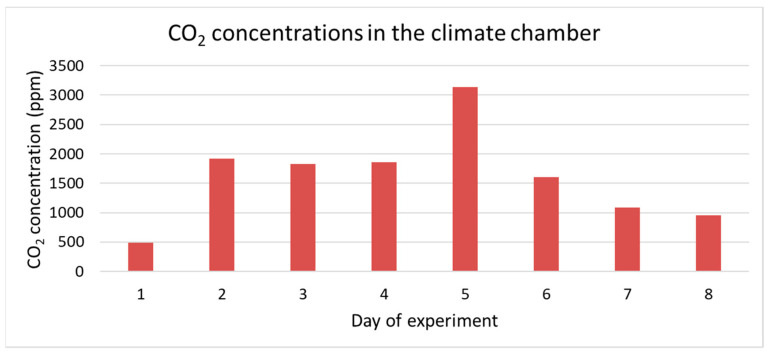
CO_2_ concentrations measured in the climate chamber.

CO_2_ concentrations increased during the first five days with a peak at five days of age, followed by a decrease during the last three days (Figure 1). The maximum measured CO_2_ concentration was 3140 ppm at day 5 of the experiment.

**Figure 2:**
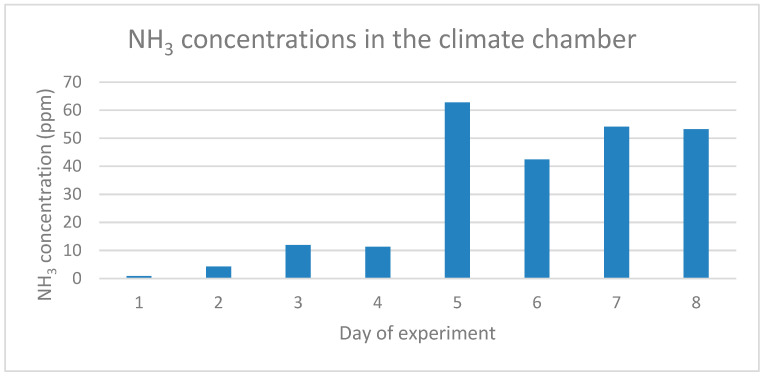
NH_3_ concentrations measured in the climate chamber.

NH_3_ concentrations were at a low level until day 4 (Figure 2). NH_3_ concentrations increased very fast to 63 ppm at day 5. NH_3_ concentration remained at a level between 40 and 60 ppm after day 5.

**Figure 3:**
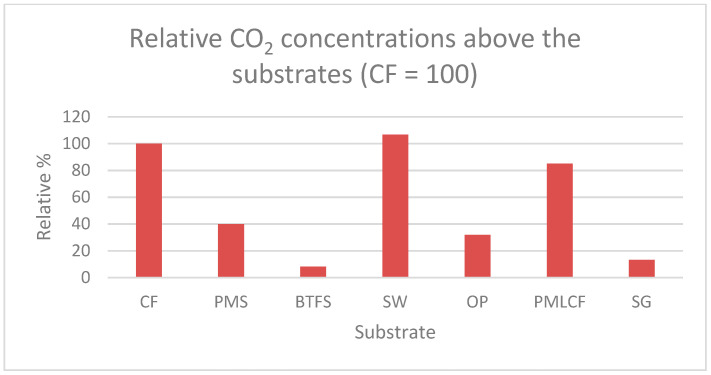
CO_2_ concentrations measured above the individual substrates at day 8.

**Figure 4:**
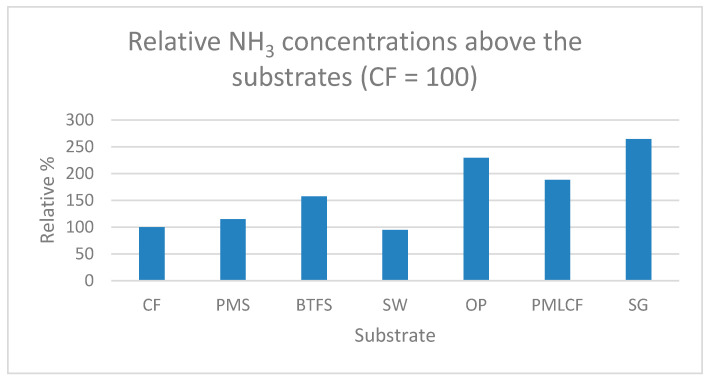
NH_3_ concentrations measured above the individual substrates at day 8.

CO_2_ and NH_3_ concentrations measured above the different substrates at day 8 are presented in Figure 3 and Figure 4, respectively, and are presented relative to the concentrations measured at CF.

The highest CO_2_ concentrations were measured at day 8 above the substrates SW, CF, PMLCF and PMS in descending order. Lowest CO_2_ concentrations were measured above the substrates OP, SG and BTFS.

The highest NH_3_ concentrations were measured at day 8 above the substrates SG, OP, PMLCF and BTFS in descending order. Lowest NH_3_ concentrations were measure above the substrates PMS, CF and SW.

## 4. Discussion

This assay investigated how seven substrates affect BSF larvae bioconversion in terms of larval growth, waste reduction and conversion efficiency of ingested substrates. The larval density was 2500 starter larvae per kilogram wet substrate and was similar in all containers because from literature it is clear that larval density affects larval growth and development [16,17]. Water was added to the substrates CF and OP to make the substrates more optimal for growth and development of BSF larvae. There is a lack of literature data on the most suitable moisture content of substrates in BSF bioconversion for effective residue separation [10] but these authors showed that the residue can be effectively separated from the insect biomass by sieving at 70% and 75% moisture content. For the current experiment, it was aimed to create a substrate containing about 65% moisture. Therefore, CF and OP substrates were mixed with water (1:1 *w*/*w*) and liquid pig manure was mixed with CF (1:1 *w*/*w*) to create PMLCF. Also, 450 mL water was added in each container with PMS because the substrate was assumed to be too dry for ingestion. The dry matter content at the start of the experiment was in all treatments around 30–35% except for OP (50.0%). The experiment ended at 8 days for the fast-growing larvae in CF, PMS, SW and PMLCF substrates because at 8 days no substrate was left at SW. First larvae on SW reached the prepupae stage at the end of the experiment. Containers with CF still contained residual substrate. Containers with PMS, BTFS, OP, PMLCF and SG had a dry top-layer and before determining the dry matter content of the remaining substrate the dry top-layer at PMS, BTFS, OP and SG was removed. The substrate below this top layer was stirred prior to sampling. Dry matter content in CF and PMLCF were above 80% at the end of the experiment and dry matter content in other substrates varied from 62% in PMS to 38% in BTFS. Dry matter content of larvae was positively correlated with growth performance. The highest dry matter content was found for larvae on SW (38%), subsequently followed by CF, PMLCF, OP, BTFS, PMS and SG (14%). In other papers, dry matter content of fresh larvae has been reported between 20% and 44% [12,18], and depends on both substrate and larval stage [19], because dry matter is higher in the later instars. Highest larval growth rate (13.4 mg/d) was found in larvae reared on SW and was higher than in larvae reared on the reference substrate CF (7.2 mg/d) and PMLCF (7.3 mg/d). The final larval weight (69 mg) on the reference substrate was in the range of larval weights obtained on a chicken feed in another experiment [13]. Larval growth rate (13.4 mg/d) of larvae fed SW containing pasta, potatoes and vegetables in the experiment was higher than the VEGFRU or FRU substrates [20]. The authors [20] studied the effect of different rearing substrates (vegetable–fruit waste–VEGFRU-celery 43.4%, oranges 28.9% and peppers 27.7%; fruit waste–FRU-apples 47.8%, oranges 15.5%, apple leftovers 13.8%, strawberries 7.1%, mandarins 4.8%, pears 4.1%, kiwis 3.4%, bananas 1.9% and lemons 1.6%; winery by-product–WIN-containing grape seeds, pulp, skins, stems and leaves; brewery by-product–BRE-Barley brewers’ grains wet) on the development of BSF larvae. Each substrate was ground with a 3 mm die meat mincer and mixed prior to placing the substrates in the containers. Larval growth rate was highest in BRE followed by FRU, VEGFRU and WIN (14 ± 0.9, 7 ± 0.7, 6 ± 1.8 and 6 ± 0.9 g/d, respectively). A large variation in larval growth and development on different substrates such as poultry feed, restraint waste, faeces, vegetables and flesh were also reported [18]. Differences in growth rate may be explained by the chemical composition of the substrates, but these were not determined in this experiment because the aim was to select the most promising substrates for growth performance. The most optimal substrates from this experiment were selected and will be tested again or mixed as test substrate to grow BSF larvae in a follow-up experiment. Waste Reduction Index (WRI) was highest on SW followed by PMLCF and both were higher than WRI on CF. Waste Reduction Index decreased subsequently further from PMS, SG, BTFS to OP. Food wastes, such as swill in the current experiment, provide good conditions for larval growth and therefore, BSF composting could be a good option [21]. Waste reduction was evaluated by growing BSF larvae on okara (soy pulp or fiber obtained from tofu production), wet brewers’ spent grains (from the beer industry), and maize distiller’s grains (from spirit or ethanol production) and a laying hen diet as a reference [22]. Waste reduction index in this experiment increased from 3.0, 3.2, 4.5 to 4.9 in the brewer’s grain, maize distillers, hen diet and okara, respectively. The WRI on CF in the current experiment was also 4.5 and the WRI on the vegetal substrates in the current experiment was 0.5, 1.2 and 1.9 for OP, BTFS and SG which is lower than in the vegetal substrates tested in the experiment of Bava et al. [22]. Waste reduction of PMS was slightly higher than the vegetal substrates in the current experiment, but pig manure still contains undigested dietary components, such as cellulose and lignin and BSF larvae cannot ingest these components [23]. The Efficiency of Conversion of Ingested substrate (ECI) was highest on SW followed by PMLCF, CF and PMS. The substrates with OP, BTFS and SG resulted in a lower ECI than other substrates. Substrates that contain a high proportion of easily available carbon, but a low content of nitrogen, such as OP or SG in this experiment, do not support larval development and thus the efficiency of the process is reduced [21]. 

The CO_2_ concentration in the climate chamber peaked at day 5 of the experiment. During the first five days CO_2_ concentrations increased followed by decrease after day 5 of the experiments whereas NH_3_ concentrations were low during the first four days of the experiment and increased very fast at five days. This course of the CO_2_ and NH_3_ concentrations during the experiment was also found in an experiment by Parodi, et al. [24]. These authors observed a clear peak in CO_2_ production between day 5 and day 6. They indicated that the drop in CO_2_ concentration observed on day 5 was caused by the physiological response of BSF larvae to either limited availability or accessibility of fresh feed. The authors concluded also that microbial metabolism in the substrate contributed to 34% of the overall CO_2_ emissions. The results demonstrate that the contribution of microbial respiration to the overall CO_2_ production during the rearing period is substantial. CO_2_ is the major gas product resulting from BSFL treatment of organic waste and is related to the respiration rate of associated microorganisms and BSF larvae, which can indirectly indicate the biodegradation rate of the substrate [25,26,27]. In the experiment of Parodi et al. [24], the NH_3_ concentration also had a defined temporal pattern. NH_3_ was produced from day 5 onwards, right after the peak of CO_2_. The timing of NH_3_ emissions might be explained by the high excretion rates of uric acid during the larval metabolic peak, followed by the microbial breakdown of uric acid into NH_4_^+^, and the subsequent volatilization of NH_3_. Considering that the occurrence and intensity of NH_3_ emissions are associated with the timing and total production of CO_2_, it is likely that NH_3_ emissions are the result of changes taking place in the substrate when larval metabolism is high (e.g., changes in temperature, moisture, pH and microbial activity) [24]. At the end of the experiment the highest CO_2_ concentrations were determined above the substrates SW, CF, PMLCF and PMS and the lowest concentrations above the substrates OP, SG and BTFS. Highest NH_3_ concentrations, however, were measured above the substrates SG, OP, PMLCF and BTFS and lowest concentrations above the substrates PMS, CF and SW. A direct link has been observed between gas concentrations and growth performances, WRI and ECI of BSF larvae. Highest CO_2_ concentrations and lowest NH_3_ concentrations have been determined at substrates resulting in the highest growth performance, WRI and ECI. The BSF larvae in the suboptimal substrates have a less efficient nitrogen metabolism, where the nitrogen is not built into insect protein and subsequently is broken down into NH_3_. These findings were confirmed by Parodi et al. [24]. Further research for a deeper understanding of this process of microbial activity in the substrates, larval metabolism and performance in different substrates varying in dry matter, chemical composition and physical properties is required.

## 5. Conclusions

Biowaste conversion of seven substrates by black soldier fly (BSF) larvae was assessed to screen the potential to upgrade these substrates into valuable resources. BSF larvae showed the highest growth rate when reared on SW. Growth rate of larvae on SW was almost twice as high as on the reference substrate CF. Other substrates with potential were PMLCF and PMS. Dry matter content of BSF larvae was highest of larvae grown on the substrates with the highest potential illustrating a direct relationship between larval development stage and dry matter content. The substrates with the highest potential resulted also in the highest WRI and ECI. Indicative measurements of CO_2_ and NH_3_ in the climate chamber demonstrated a peak in CO_2_ concentration at day 5 and after the peak in CO_2_ concentration the NH_3_ concentration increased. Highest CO_2_ concentrations and lowest NH_3_ concentrations were measured above the substrates SW, CF, PMLCF and PMS which are the substrates that resulted in the highest growth performance, WRI and ECI. The relation between dry matter and chemical composition of the substrates and larval development and growth performance will be further studied in follow-up experiments.

## Figures and Tables

**Table 1:** Quantity of substrate, number of larvae per container and available substrate per larva.

Substrate	Quantity of Wet Substrate (kg)	Number of BSF Larvae	Substrate per BSF Larva (g)
Chicken feed (CF; reference diet)	10.0	24,995	0.4
Pig manure solid (PMS)	6.8	16,910	0.4
Betafert^®^ solid (BTFS)	10.0	24,892	0.4
Swill (SW)	9.9	24,805	0.4
Olive pulp (OP)	10.0	25,021	0.4
Pig manure liquid mixed with CF (PMLCF)	9.7	24,289	0.4
Silage grass (SG)	4.9	12,374	0.4

**Table 2:** Dry matter content of the substrates ^1^ at start and end of the experiment and dry matter content of BSF larvae at end of the experiment.

Dry Matter (%)	CF	PMS	BTFS	SW	OP	PMLCF	SG	SEM	*p*-Value
Substrate start	40 ^b^	32 ^d^	30 ^e^	28 ^f^	57 ^a^	38 ^c^	30 ^e^	0.1	<0.001
Substrate end	81 ^a^	62 ^b^	38 ^d^	47 ^c^	66 ^b^	83 ^a^	43 ^cd^	1.6	<0.001
BSF larvae end	33 ^b^	21 ^d^	17 ^e^	38 ^a^	27 ^c^	32 ^b^	14 ^e^	0.6	<0.001

^a–f^ Means with different letters in a row are significantly different (*p* < 0.05). ^1^ Chicken feed (CF; reference diet), Pig manure solid (PMS), Betafert^®^ solid (BTFS), Swill (SW), Olive pulp (OP), Pig manure liquid mixed with CF (PMLCF), Silage grass (SG).

**Table 3:** Growth performance (fresh live weight), Waste Reduction Index (dry matter) and Efficiency of Conversion of Ingested substrate (dry matter) of BSF larvae grown on seven different substrates ^1^.

Parameter	CF	PMS	BTFS	SW	OP	PMLCF	SG	SEM	*p*-Value
Day of harvest (d)	8	8	11	8	11	8	11	-	-
BSF larvae weight start (mg)	11	11	11	11	11	11	11	-	-
BSF larvae weight end (mg)	69 ^b^	37 ^c^	18 ^d^	119 ^a^	13 ^d^	70 ^b^	19 ^d^	1.6	<0.001
Larval growth rate (mg/d)	7.2 ^b^	3.2 ^c^	0.6 ^d^	13.4 ^a^	0.2 ^d^	7.3 ^b^	0.7 ^d^	0.17	<0.001
Waste Reduction Index (DM g/d)	4.5 ^c^	3.0 ^d^	1.2 ^ef^	10.6 ^a^	0.5 ^f^	5.4 ^b^	1.9 ^e^	0.15	<0.001
Efficiency of Conversion of Ingested substrate (g:g DM)	0.34 ^abc^	0.31 ^abc^	0.20 ^cd^	0.46 ^a^	0.26 ^bcd^	0.37 ^ab^	0.14 ^d^	0.027	<0.001

^a–f^ Means with different letters in a row are significantly different (*p* < 0.05). ^1^ Chicken feed (CF; reference diet), Pig manure solid (PMS), Betafert^®^ solid (BTFS), Swill (SW), Olive pulp (OP), Pig manure liquid mixed with CF (PMLCF), Silage grass (SG).

## Data Availability

Data is available upon request from the corresponding author and pending agreement by co-authors.
